# Joint genome-wide association study of progressive supranuclear palsy identifies novel susceptibility loci and genetic correlation to neurodegenerative diseases

**DOI:** 10.1186/s13024-018-0270-8

**Published:** 2018-08-08

**Authors:** Jason A. Chen, Zhongbo Chen, Hyejung Won, Alden Y. Huang, Jennifer K. Lowe, Kevin Wojta, Jennifer S. Yokoyama, Gilbert Bensimon, P. Nigel Leigh, Christine Payan, Aleksey Shatunov, Ashley R. Jones, Cathryn M. Lewis, Panagiotis Deloukas, Philippe Amouyel, Christophe Tzourio, Jean-Francois Dartigues, Albert Ludolph, Adam L. Boxer, Jeff M. Bronstein, Ammar Al-Chalabi, Daniel H. Geschwind, Giovanni Coppola

**Affiliations:** 1 Interdepartmental Program in Bioinformatics, University of California, Los Angeles, CA 90095 USA; 2 Maurice Wohl Clinical Neuroscience Institute, Department of Basic and Clinical Neuroscience, King’s College London, London, SE5 9RX UK; 3 Program in Neurogenetics, Department of Neurology and Department of Human Genetics, David Geffen School of Medicine at UCLA, Los Angeles, CA 90095 USA; 4 Semel Institute for Neuroscience and Human Behavior, University of California, Los Angeles, CA 90095 USA; 5 Memory and Aging Center, Department of Neurology, University of California, San Francisco, CA 94158 USA; 6 BESPIM, CHU-Nîmes, Nîmes, France; 7 Dept Pharmacologie Clinique, Pitié-Salpêtrière Hospital, AP-PH, Paris, France; 8 Pharmacology UPMC-Paris VI, Universite Paris-Sorbonne, Paris, France; 9 Trafford Centre for Biomedical Research, Brighton and Sussex Medical School, University of Sussex, Falmer, Brighton, UK; 10 Medical Research Council Social, Genetic and Developmental Psychiatry Centre, and Department of Medical and Molecular Genetics, King’s College London, London, SE5 8AF UK; 11 William Harvey Research Institute, Barts and The London School of Medicine and Dentistry, Queen Mary University of London, Charterhouse Square, London, EC1M 6BQ UK; 12 Univ. Lille, Inserm, CHU Lille, Institut Pasteur de Lille, U1167 - RID-AGE - Risk Factor and molecular determinants of aging diseases, Labex-Distalz, F-59000 Lille, France; 13 Univ. Bordeaux, Inserm, Bordeaux Population Health Research Center, UMR 1219, CHU Bordeaux, F-33000 Bordeaux, France; 14 Department of Neurology, University of Ulm, Oberer Eselsberg, Ulm, Germany; 15 Departments of Psychiatry and Neurology, David Geffen School of Medicine, University of California, Los Angeles, 695 Charles E Young Dr. South, Gonda Bldg, Rm 1524, Los Angeles, CA 90095 USA

**Keywords:** Genome-wide association study, Progressive supranuclear palsy, Neurodegeneration

## Abstract

**Background:**

Progressive supranuclear palsy (PSP) is a rare neurodegenerative disease for which the genetic contribution is incompletely understood.

**Methods:**

We conducted a joint analysis of 5,523,934 imputed SNPs in two newly-genotyped progressive supranuclear palsy cohorts, primarily derived from two clinical trials (Allon davunetide and NNIPPS riluzole trials in PSP) and a previously published genome-wide association study (GWAS), in total comprising 1646 cases and 10,662 controls of European ancestry.

**Results:**

We identified 5 associated loci at a genome-wide significance threshold *P* < 5 × 10^− 8^, including replication of 3 loci from previous studies and 2 novel loci at 6p21.1 and 12p12.1 (near *RUNX2* and *SLCO1A2*, respectively). At the 17q21.31 locus, stepwise regression analysis confirmed the presence of multiple independent loci (localized near *MAPT* and *KANSL1*). An additional 4 loci were highly suggestive of association (*P* < 1 × 10^− 6^). We analyzed the genetic correlation with multiple neurodegenerative diseases, and found that PSP had shared polygenic heritability with Parkinson’s disease and amyotrophic lateral sclerosis.

**Conclusions:**

In total, we identified 6 additional significant or suggestive SNP associations with PSP, and discovered genetic overlap with other neurodegenerative diseases. These findings clarify the pathogenesis and genetic architecture of PSP.

**Electronic supplementary material:**

The online version of this article (10.1186/s13024-018-0270-8) contains supplementary material, which is available to authorized users.

## Background

Tau pathology is a prominent hallmark of neurodegenerative diseases, including Alzheimer’s disease (AD) and frontotemporal dementia (FTD). Progressive supranuclear palsy (PSP) is a relatively pure tauopathy associated with parkinsonism - dementia, characterized by pathological tau aggregation and a clinical syndrome of postural instability, falls, and supranuclear ophthalmoplegia [1]. It shares symptomatic and neuropathologic overlap with a large group of diseases, that are collectivity known as “tauopathies” due to characteristic tau deposits; however, compared to these diseases, PSP appears to be more clinically, neuropathologically, and genetically homogenous [2–4]. Notably, the clinical syndrome has high correlation with the neuropathology [5]. These characteristics have thrust PSP into a central role for studying neurodegeneration, enabling clinical trials of a relatively homogenous patient population with potentially more uniform response to treatment. Therefore, PSP has become a target of intense clinical research [6, 7]. While the disease shares neuropathological overlap with other tauopathies, the polygenic genetic correlation with other neurodegenerative diseases remains to be clarified.

The major known genetic risk factor is an extended H1 haplotype on chromosome 17q21.31, which includes *MAPT* (the gene encoding the tau protein), and is homozygous in almost all PSP patients [2]. Other risk factors identified include genome wide significant associations at loci near *MAPT*, *MOBP*, *STX6*, and *EIF2AK3*, suggesting a strong contribution of common variation in its genetic architecture [8]. We reasoned that the inclusion of additional cases and controls could increase the statistical power for genome-wide association, potentially yielding novel loci that could provide insight into the molecular mechanisms of PSP and other more common tauopathies.

## Methods

### Cohort

Three cohorts of primarily European ancestry were included in the study – “UCLA”, a combination of 349 PSP patients and 130 controls from the UCSF Memory and Aging Center [2, 9] and the Allon Therapeutics Davunetide trial [6]; “NNIPPS”, a group of 341 PSP patients from the Neuroprotection and Natural History in Parkinson Plus Syndromes (NNIPPS) trial [7] and the Blood Brain Barrier in Parkinson Plus syndromes (BBBIPPS) study; and “Hoglinger”, 1112 PSP patients from a previously published GWAS [8]. The UCLA cohort was divided into two, because of differences in genotyping platform: “UCLA Omni 2.5” and “UCLA HumanCore”. Further details are available in the Supplementary Methods.

### Genotyping

Genotyping in the UCLA study cohort was performed as a prelude to whole-genome sequencing, and was performed by Illumina (using the Illumina HumanOmni 2.5 Array) and the New York Genome Center (using the Illumina HumanCore Array). Genotyping calls were made using the Illumina GenomeStudio software.

### Public datasets

Genotypes from the Hoglinger et al. GWAS [8] (cases only – no controls) were obtained from the NIAGADS database. Out-of-sample controls were obtained from dbGAP Authorized Access to match each genotyping platform. In total, for the HumanOmni 2.5 M platform, we obtained 2364 subjects; for the OmniExpress platform, 870 subjects; and for the HumanQuad 660 W platform, 8756 subjects. For the Illumina HumanQuad 660 W Array (Hoglinger et al. study), we used phs000103.v1.p1 “Genome-Wide Association Studies of Prematurity and Its Complications”, phs000289.v1.p1 “National Human Genome Research Institute (NHGRI) GENEVA Genome-Wide Association Study of Venous Thrombosis”, phs000188.v1.p1 “Vanderbilt Genome-Electronic Records (VGER) Project: QRS Duration”, phs000203.v1.p1 “A Genome-Wide Association Study of Peripheral Arterial Disease”, phs000237.v1.p1 “Northwestern NUgene Project: Type 2 Diabetes”, phs000234.v1.p1 “Group Health/UW Aging and Dementia eMERGE study”, and phs000170.v1.p1 “A Genome-Wide Association Study on Cataract and HDL in the Personalized Medicine Research Project Cohort”. For the Illumina HumanOmni 2.5 Array (UCLA – this study, and NNIPPS study), we used phs000371.v1.p1 “Genetic Modifiers of Huntington’s Disease”, phs000429.v1.p1 “NEI Age-Related Eye Disease Study (AREDS) - Genetic Variation in Refractive Error Substudy”, and phs000421.v1.p1 “A Genome-Wide Association Study of Fuchs’ Endothelial Corneal Dystrophy (FECD)”. For the Illumina HumanCore Array (UCLA – this study), we used the WTCCC2 cohort, which was typed on the related Illumina OmniExpress Array. Subjects with an ascertained phenotype (e.g., disease) were removed. More detailed information regarding these datasets is available in Additional file 1: Table S1.

### Data preprocessing

Genotypes for all datasets were converted to the forward strand, and converted into coordinates based on the hg19 reference sequence using UCSC liftOver [10]. The genotypes were then merged and pre-processed according to platform. Determination of cryptic relatedness (pairwise proportion IBD, PI-HAT > 0.2), sample missingness (> 0.05), genotype missingness (> 0.05), Hardy-Weinberg equilibrium *p*-value (< 10^−5^), and sex-matching was performed in PLINK v1.90b3.28 [11] and used to quality-control (QC) samples using standard parameters [12]. Ancestry was predicted by multidimensional scaling based on raw Hamming distances, implemented in PLINK. Only samples of presumed European ancestry that clustered with known Europeans from the HapMap3 cohort [13] were included. Preprocessing steps are further elaborated in Additional file 2: Figure S1.

### Imputation

Imputation was performed separately for each genotyping platform using the IMPUTE v2.3.2 algorithm [14]. Prephasing of chromosomes using the Segmented HAPlotype Estimation & Imputation Tool (SHAPEIT) v2.r837 was performed as previously described [15, 16]. IMPUTE2 was run on the prephased haplotypes using the 1000 Genomes Project Phase 3 reference in non-overlapping 5 megabase chunks with a 250 kilobase buffer and an effective population size of 20,000. Imputed variants with an imputation genotype probability < 0.9, missingness > 0.05, or minor allele frequency < 0.01 were removed, and genotypes across platforms were merged. Cryptic relatedness across cohorts was assessed, and related/duplicated samples were removed.

### Association

Association was performed using a linear mixed model to correct for population structure, using BOLT-LMM [17]. The genotyping platform was used as a categorical covariate. The standard infinitesimal model *p*-values were chosen for downstream anaylsis. Odds ratios were calculated as exp. (beta). Because some of the individual cohort sizes violate the large sample size assumptions of BOLT-LMM, odds ratios for association (for individual cohorts) were computed using a logistic regression model in PLINK, using the first 5 eigenvectors, derived from Principal Component Analysis (PCA), as covariates. Power calculations were performed using the Genetic Power Calculator [18], assuming a variant with risk allele frequency of 0.5 and relative risk of 1.3 in an additive genetic model, a disease with a prevalence of 10 in 100,000, and a p-value threshold of 5 × 10^− 8^, using a genotypic, 2 df case-control test. QQ and Manhattan plots were constructed using the R package “qqman” [19]. Forest plots were constructed using the R package “metafor” [20]. The genomic inflation factor λ was computed with PLINK. Correction of the genomic inflation factor to an equivalent sample size of 1000 cases and 1000 controls was performed as previously described [21]. To control for the extended haplotype on chr17q21 and to identify independent association signals, we performed association as before, but including the haplotype (tagged by the SNP rs1560310) [22] as a covariate.

### Proportion variance in liability explained

The explained variance in liability at each of the genome-wide significant loci was calculated according to the method of So et al., which requires the frequency of the risk allele, the relative risk of the heterozygous genotype, the relative risk of the homozygous risk genotype, and the prevalence of the disease in the population [23]. The allele frequencies were calculated from the control population of the joint genotyping cohort. Relative risks were approximated with the corresponding odds ratios, which converges to relative risk when the prevalence of disease is rare. Genotypic odds ratios were estimated by assuming an additive model. The prevalence of PSP was estimated at 6.5 per 100,000 in accordance with prior epidemiological studies [24, 25]. The genome-wide polygenic variance in liability explained was calculated using GCTA v1.24.7 [26]. The genetic relationship matrix was calculated chromosome-by-chromosome and then re-combined. The first 5 principal components were calculated and used as covariates for restricted maximum likelihood (REML) analysis.

### Prediction of gene expression differences associated with PSP-associated SNPs

Genetic associations with PSP may be due to genetic control of gene expression. We used TWAS to predict differential gene expression in PSP from the joint analysis summary statistics, integrating paired genotyping and gene expression data from the GTEx Consortium [27]. Correcting for approximately 5000 effective independent tests per brain region (taking into account 5483 genes with significantly heritable weights and the interdependence of gene expression, particularly across tissues), the significance threshold was set at *P* < 1 × 10^− 5^.

### Credible set of causal variants at PSP GWAS loci

A credible set (potential causal variants) was identified at each of total of seven genome-wide significant loci identified in this study using the CAusal Variants Identification in Associated Regions (CAVIAR) software package [28]. Because of the extended linkage disequilibrium patterns in the chromosome 17q21.31 haplotype region, causal variants were not identified at this associated locus. Within each of the selected loci, the SNP with the minimum joint association *p*-value was chosen as the index SNP, and variants with p-value < 10^− 5^ and in LD (r^2^ > 0.6) with the index SNP were input into CAVIAR. The CAVIAR-identified credible set contains potential causal variants (with a confidence level of 95% under the statistical model) that could explain the association at each locus.

### Identification of genes linked to credible SNPs with chromatin interaction data

Genetic variation can result in changes to the coding sequence of a gene (e.g., nonsense and missense variants) or can regulate the gene’s expression (e.g., by affecting transcription factor binding in promoter or enhancer regions). We first identified credible SNPs as “functional” (stopgain variant, frameshift variant, splice donor variant, nonsense-mediated decay transcript variant, or missense variant). Of the remaining credible SNPs, we identified those in the promoter region of a gene, defined as the range 2 kb upstream to 1 kb downstream relative to the transcription start site (TSS). Finally, the remaining credible SNPs were considered possible regulatory variants and tested for short- or long- range interaction with other regions of chromatin to identify potential downstream target genes. The interactions were determined by Hi-C experiments in IMR90 and embryonic stem cells from public data [29, 30], and fetal brain germinal zone (ventricular and subventricular zone) and cortical plate (intermediate zone and marginal zone) from our group [31].

### Genetic correlation with neurodegenerative diseases

Genetic correlation was assessed from GWAS summary statistics using the Linkage Disequilibrium Score Regression method (LDSC) [32]. Summary statistics were filtered by only considering SNPs that overlap with the HapMap3 reference panel. Refer to the Supplementary Methods for further details.

### Data availability

Full and imputed genotyping results from the UCLA and NNIPPS cohorts will be made available on the NIAGADS database.

## Results

We analyzed subjects from three GWAS cohorts, including 1) a multi-center cohort [2, 6] in whom we performed genotyping using the Illumina HumanOmni2.5 BeadChip and the Illumina HumanCore BeadChip (“UCLA”); 2) patients from centers in France, Germany, and the United Kingdom as part of the Neuroprotection and Natural History in Parkinson Plus Syndromes (NNIPPS) study, a double-blind randomized placebo-controlled clinical trial of riluzole [7], genotyped with the Illumina HumanOmni2.5 BeadChip (“NNIPPS”), and 3) a cohort of autopsy-proven cases from a previously published [8] GWAS (“Hoglinger”). A more detailed description of the cohorts is provided in Additional file 1: Table S1. We combined each cohort with platform-matched, out-of-sample controls from dbGAP (Additional file 1: Table S1). Stringent QC – excluding SNPs that had low genotype call rates (< 0.95) or did not follow Hardy-Weinberg equilibrium, and excluding subjects with low sample call rate (< 0.95), non-European ancestry, incompatible sex, cryptic relatedness, or duplication across cohorts (Additional file 2: Figure S1) – was applied to each cohort (including platform-matched controls). We then imputed variants implementing IMPUTE2 [14] using the 1000 Genomes Phase 3 Reference Panel to estimate genotypes at more than 77,000,000 SNPs. Imputed variants with imputation quality scores (*r*^*2*^ < 0.9) or low minor allele frequency (< 0.01) were filtered, and genotypes across all cohorts were combined in a joint analysis. In total, we examined 6,419,662 SNPs in 1646 PSP cases and 10,662 controls.

Association was initially performed using the 616 cases represented from the UCLA and NNIPPS cohorts. Genome-wide significant association was detected at loci near *MAPT* and largely corresponded to the haplotype region (lead SNP rs79730878, *p* = 5.4 × 10^− 45^; Additional file 1: Table S2). Other top associations were found at loci near *MOBP*, *STX6*, *SEMA4D*, *DDX27*, and *SP1*, though these did not reach genome-wide significance.

**Fig. 1:**
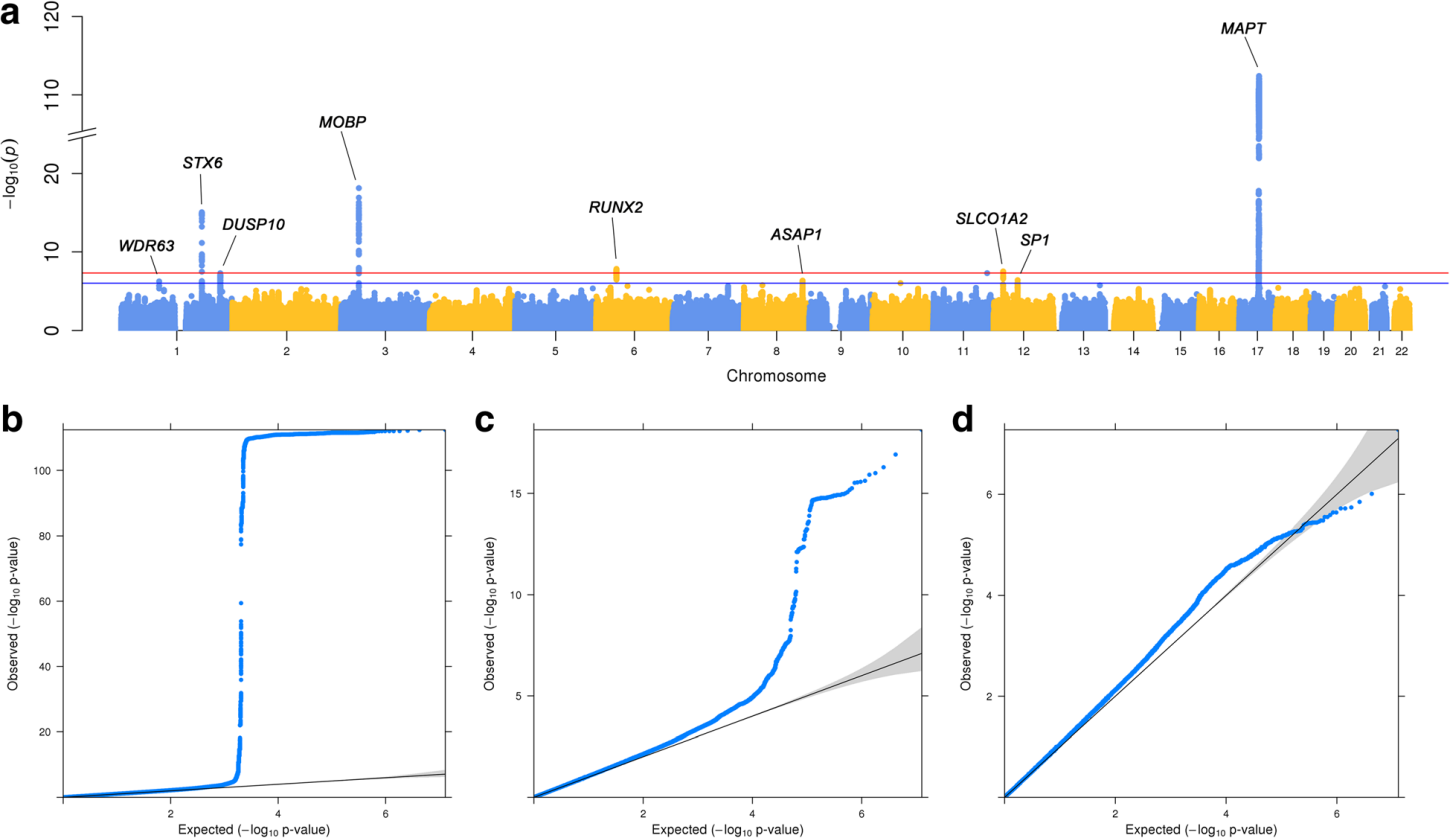
Genome-wide SNP association in the joint analysis. **a** Manhattan plot indicating the SNP association *P* values. The vertical axis displays the strength of association (−log10 *P* value) as a function of genomic position, with alternating colors for sequential chromosomes. Genome-wide significant and suggestive loci are labeled with the nearest gene symbol. The thresholds for significant (*P* < 5 × 10^− 8^, red horizontal line) and suggestive (*P* < 1 × 10^− 6^, blue horizontal line) associations are shown. **b-d** Quantile-quantile plots for: **b** all SNPs, including the strongly associated extended haplotype on chromosome 17; **c** SNPs excluding chromosome 17; and **d** SNPs excluding genome-wide significant and suggestive loci. The 95% confidence interval for the expected distribution of *p*-values is shaded

To increase statistical power, we combined all three cohorts in a joint analysis framework. We estimated that this combined cohort had 90% power to detect association of a variant with allele frequency of 0.5 and relative risk of 1.3. For a cohort of the sample size of that in a previous PSP GWAS from Hoglinger et al., the power to detect association was only 33%. In the primary analysis, we assessed the genome-wide association between the genotype at each SNP and case-control status using a linear mixed model to correct for population stratification. The genomic inflation factor λ for the joint analysis was 1.05; for the UCLA-Omni2.5, UCLA-HumanCore, NNIPPS, and Hoglinger cohorts, λ was 1.03, 1.02, 1.11, and 1.11, respectively (Fig. 1, Additional file 2: Figure S2 and S3). We considered the joint inflation factor to be acceptable in the setting of a relatively large joint analysis sample size [33]. Scaled for sample size, the adjusted genomic inflation factor λ_1000_ was 1.02.

**Fig. 2:**
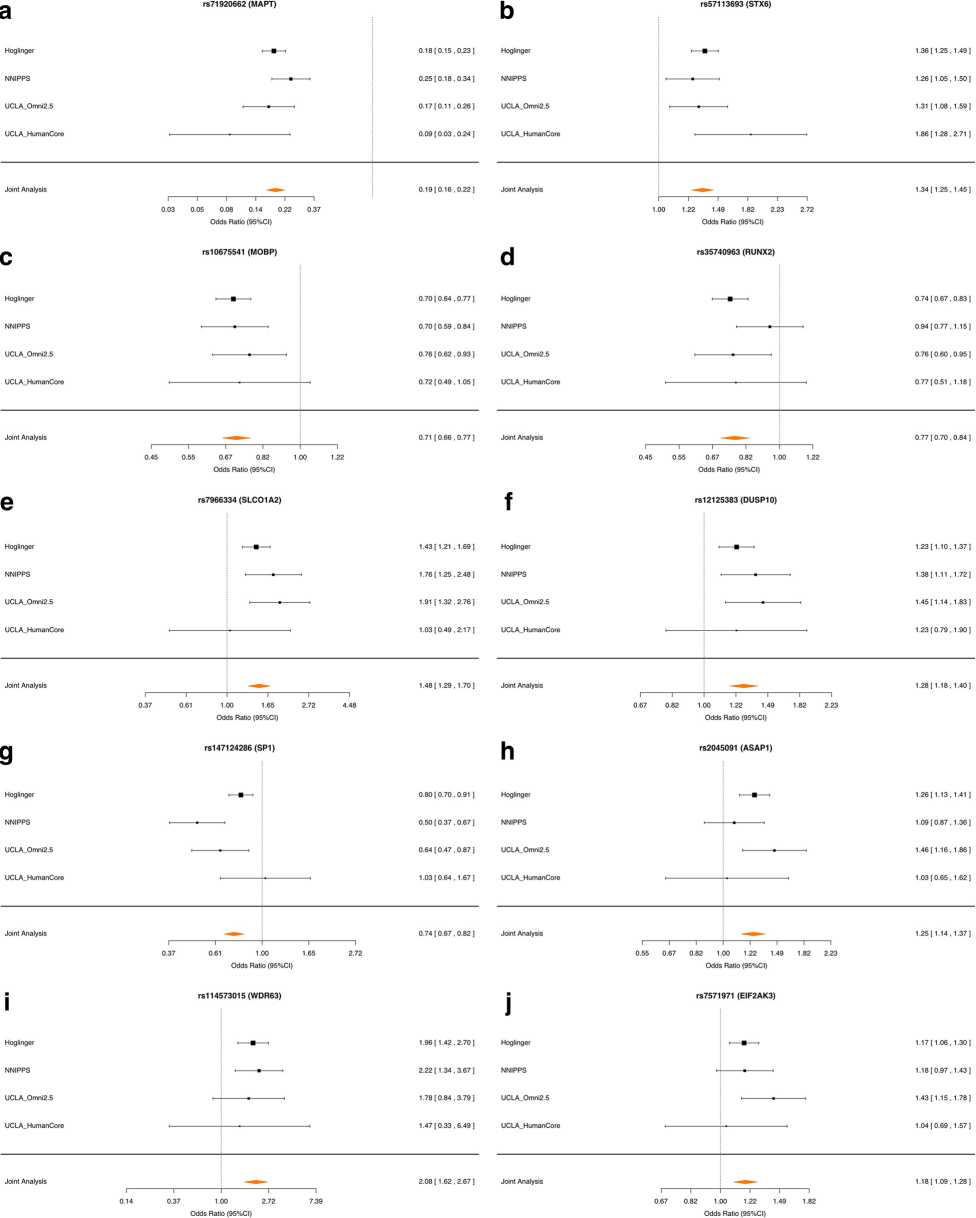
Forest plots showing association across each individual cohort for selected SNPs. A total of six genome-wide significant loci were identified, with representative SNPs: **a** rs71920662 in 17q21.31, near *MAPT*; **b** rs57113693 in 1q25.3, near *STX6*; **c** rs10675541 in 3p22.1, near *MOBP*; **d** rs35740963 in 6p21.1, near *RUNX2*; and **e** rs7966334 in 12p12.1, near *SLCO1A2.* An additional four suggestive loci were also identified: **f** rs12125383 in 1q41, near *DUSP10* in an intergenic region; **g** rs147124286 in 12q13.13, near *SP1*; **h** rs2045091 in 8q24.21, near *ASAP1*; and **i** rs114573015 in 1p22.3, near *WDR63*. **j** Additionally, a previously reported GWAS SNP rs7571971 in 2p11.2, near *EIF2AK3*, was not identified as genome-wide significant in the joint analysis

The results of the joint analysis genome-wide association are shown in Fig. 1 and Additional file 2: Figure S4. SNPs at 5 loci, in cytobands 17q21.31 (in an extended haplotype containing *MAPT*, lead SNP rs71920662, odds ratio OR = 0.19, *p* = 3.9 × 10^− 113^), 3p22.1 (within *MOBP*, rs10675541, OR = 0.71, *p* = 7.2 × 10^− 19^), 1q25.3 (within *STX6*, rs57113693, OR = 1.3, *p* = 8.7 × 10^− 16^), 6p21.1 (within *RUNX2*, rs35740963, OR = 0.77, *p* = 1.8 × 10^− 8^), and 12p12.1 (within *SLCO1A2*, rs7966334, OR = 1.5, *p* = 3.2 × 10^− 8^), reached genome-wide significance (*p* < 5 × 10^− 8^) (Additional file 1: Table S2, Additional file 2: Figure S5). An additional SNP reported in a previous GWAS [8], rs7571971, was also analyzed. Although this SNP did not reach genome-wide significance in the joint analysis (OR = 1.18, *p* = 2.7 × 10^− 5^), the direction of the association was consistent with the previous association in each cohort. In order to decrease the likelihood that the results were influenced by population stratification, we assessed association at the loci in each of the study cohorts (Fig. 2). Associations at the lead SNPs in each of the regions were consistent across the three most well-powered study cohorts (Hoglinger, NNIPPS, and UCLA Omni2.5) while in general, the HumanCore subset of the UCLA cohort was underpowered to detect association. An additional 4 loci demonstrated suggestive association (1 × 10^− 6^ < *P* < 5 × 10^− 8^), in 1q41 (intergenic, near *DUSP10*, rs12125383, OR = 1.28, *p* = 5.3 × 10^− 8^), 12q13.13 (within *SP1*, rs147124286, OR = 0.74, *p* = 4.1 × 10^− 7^), 8q24.21 (within *ASAP1*, rs2045091, OR = 1.25, *p* = 4.7 × 10^− 7^), and 1p22.3 (near *WDR63* and *MIR4423*, rs114573015, OR = 2.1, *p* = 5.9 × 10^− 7^) (Additional file 1: Table S3). Overall, the genome-wide significant loci explained a combined 5.9% of the variance in heritable liability of PSP (Additional file 1: Table S4). The locus tagging the chr17q21 haplotype surrounding MAPT contributed the majority (5.0%), while new loci contributed an additional 0.2% of the total liability. Using a polygenic model implemented in GCTA [26], the entire set of genotyped SNPs explains 9.4 ± 0.8% (estimate±standard error) of the variance on the liability scale, suggesting that many loci are yet to be found.

The association between PSP and the chr17q21 haplotype (H1/H2) has been widely characterized, but independent SNPs in the chr17q21 region may also contribute to disease susceptibility. To test this, we performed linear regression, taking haplotype as a covariate. Additionally, we identified subjects that were homozygous for the risk allele (H1/H1), and performed association in this subset of patients. Both approaches identified similar independent associations from the H1 haplotype in the 17q21.31 region, with the most significant SNPs at rs8078967 (*P* = 1.9 × 10^− 14^) and rs9904290 (*P* = 8.9 × 10^− 12^) in the haplotype-regressed and H1/H1 only datasets, respectively (Additional file 2: Figure S5). These SNPs did not appear to be in strong linkage disequilibrium with a previously reported SNP association, rs242557 [8] (*r*^*2*^ = 0.008 and 0.007 in the 1000 Genomes Project data – EUR super-population, respectively) that was filtered from this dataset in variant QC; however, they were highly correlated with each other (*r*^*2*^ = 0.996). Additionally, both variants and rs242557 are within the first intron of the *MAPT* gene.

To further understand how variation at each of the loci contributes to disease risk, we assessed the functional consequences of significant SNPs. We first identified a set of potential causal SNPs using the CAVIAR method, which identifies a “credible set” of SNPs that encompasses those likely to be causal [28]. The 17q21.31 locus was excluded from the analysis because of its unusual, long-range linkage disequilibrium pattern. In some loci, potentially causal coding variants were identified (in genome-wide significant loci, at 6p21.1, in *RUNX2*, and at 12p12.1, in *SLCO1A2*; and in suggestive loci, at 8q24.21, in *ASAP1*, and at 12q13.13, in *AMHR2*; Additional file 1: Table S5). Other SNPs in the credible set fell within regulatory regions; we identified the gene associated with each SNP using data from Hi-C experiments, mapping chromosome conformation patterns on a genome-wide scale from four human cell types (IMR-90 fetal lung fibroblasts, embryonic stem cells, fetal brain, and fetal brain germinal zone). Each potential regulatory SNP in the credible set was then associated with genes in close proximity by chromosomal conformation, yielding potential downstream causal genes (Additional file 1: Table S5).

To supplement the mapping information from Hi-C, we also identified the functional consequences of GWAS hits using the TWAS method to predict genes that may be affected by risk alleles [27]. TWAS estimates gene expression values using paired reference transcriptome/genotyping datasets (e.g., for expression quantitative trait loci - eQTL studies) and genotype information from summary statistics, and predicts differential expression between cases and controls. Using reference data from the GTEx Consortium, TWAS predicted the effect of gene expression from the risk haplotypes in multiple tissues. At a threshold of *P* < 1 × 10^− 5^, we identified a number of genes that were called as differentially expressed (Additional file 1: Table S6). As expected due to the length and lack of recombination in the region, most of these genes (17) clustered around the chromosome 17 haplotype. Notably, *MAPT* (within the associated 17q21.31 locus) was among the genes predicted to be differentially expressed, as well as *STX6* (within the associated 1q24 locus), *SP1* (within the suggestive 12q13.13 locus), *SKIV2L* (within 6p21.33, nearby the associated 6p21.1 locus), and *RPSA* (within the associated 3p22.1 locus). Other genes that were pinpointed outside of association regions were *CEP57* (in 11q21) and *RPS6KL1* (in 14q24.3).

**Fig. 3:**
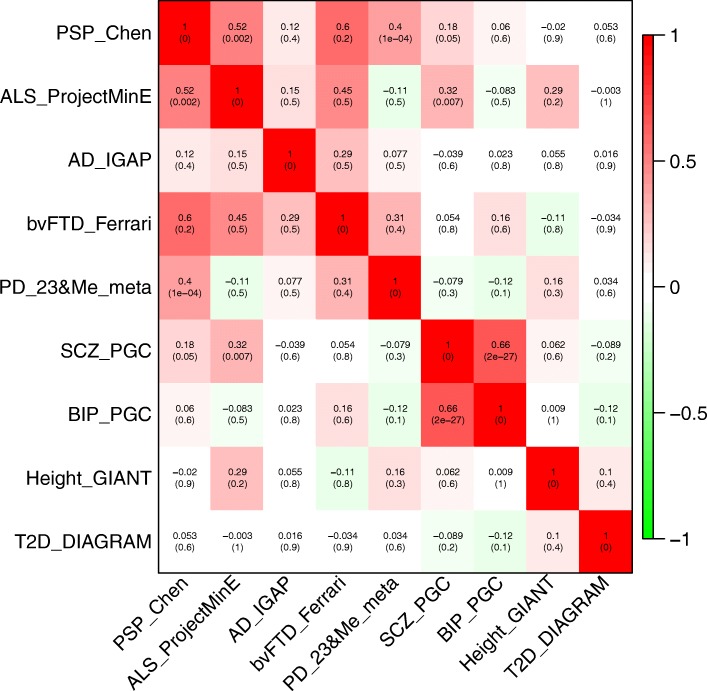
Heatmap of genetic correlation between GWAS summary statistics for neurodegenerative diseases (PSP – progressive supranuclear palsy, ALS – amyotrophic lateral sclerosis, AD – Alzheimer’s disease, bvFTD – behavioral variant frontotemporal dementia, PD – Parkinson’s disease), calculated by LDSC. GWAS for non-neurodegenerative phenotypes (SCZ – schizophrenia, BIP – bipolar disorder, height, and T2D– type 2 diabetes mellitus) are also included for comparison. In each cell, the genetic correlation coefficient (and *P* value in parentheses) is shown. Phenotypes that share a common polygenic background are positively correlated

The strong neuropathological overlap of PSP with other tauopathies suggests that genetic overlap may exist. Using the LDSC software [32], we assessed genetic overlap of PSP with other neurodegenerative diseases, including AD, behavioral variant FTD (bvFTD), Parkinson’s disease (PD), and amyotrophic lateral sclerosis (ALS), by using GWAS summary statistics. As controls, we included summary statistics from GWAS for heritable, non-neurodegenerative diseases of brain (schizophrenia and bipolar disorder), a quantitative trait (height), and a non-brain disease (type 2 diabetes) (for further details, refer to the Supplementary Methods). Each of these traits was shown to be heritable. Statistically significant genetic correlations were identified for PD (*P* = 9.7 × 10^− 5^) and ALS (*P* = 1.8 × 10^− 3^), but not for non-neurodegenerative disease control GWAS (Fig. 3).

## Discussion

As a prototypical tauopathy, insight into PSP susceptibility alleles can help to illuminate the downstream molecular effects of tau pathology, which is a major component of many common neurodegenerative diseases. Altogether, from a joint analysis of three disease cohorts, we have found 2 novel genome-wide significant susceptibility loci in PSP and replicated 3 previously reported loci. Of the loci identified in this study, three (within *MAPT*, *MOBP*, and *STX6*) were reported significant in a previous GWAS [8].

In the *MAPT* region, a third independent association was identified, also in *MAPT* intron 1, speculatively suggesting important regulatory functions in this region; however, no effects in differential expression have been uncovered. Overall, the mechanisms of the *MAPT* associations have been unclear. The larger H1/H2 haplotype appears to affect splicing at *MAPT* exon 3 but not overall tau expression [34]; while other *MAPT* variants, such as rs242557, may affect tau expression in some tissues [35], the effect is not robust in brain tissue. The additional association identified here may provide an orthogonal point of investigation into this curious region.

An additional locus near the *EIF2AK3* gene (encoding PERK, a key component of the unfolded protein response) was also previously identified; however, the reported SNP did not reach genome-wide significance in this joint analysis or in the new “Hoglinger” cohort (using different controls).

We also identified 2 novel genome-wide significant susceptibility loci at 6p21.1 and 12p12.1 (near *RUNX2* and *SLCO1A2*, respectively). At 6p21.1, we identified a lead SNP as well as several coding SNPs in the credible set within *RUNX2*, a gene thought to be a transcriptional factor involved in regulation of osteoblastic differentiation [36]. While seemingly unrelated to PSP, a curious number of neurodegeneration-related genes are also involved in bone diseases (e.g. *TREM2*, which has been linked to AD and Nasu-Hakola disease [37, 38], and *VCP*, linked to amyotrophic lateral sclerosis and Paget’s disease of bone [39, 40]). At 12p12.1, we identified a lead SNP and credible set coding SNPs within *SLCO1A2*, a transporter present (among other places) at the blood-brain barrier, where it regulates solute trafficking [41]. An additional four loci (near the genes *DUSP10*, *SP1*, *ASAP1*, and *WDR63*/*MIR4423*) were suggestive of association, but did not reach genome-wide significance. While this study raises the possibility of involvement of these genes in PSP pathogenesis, further fine-mapping and functional studies are needed to confirm their possible roles.

Our results also implicate possible alternative causal genes in previously reported genome-wide significant loci. At 3p22.1, the gene closest to the GWAS lead SNP was reported as *MOBP*. This locus has previously been implicated in differential expression of the SLC25A38/appoptosin gene, which may regulate tau cleavage [42]. Using Hi-C, we have identified chromatin interactions with *MYRIP* and *EIF1B* that could also explain this association. Similarly, at 1q25.3, the gene closest to the GWAS lead SNP was *STX6*; by Hi-C, we have also identified *XPR1* as a possible candidate gene. Interestingly, our group has previously demonstrated *XPR1* mutations in primary familial brain calcification [43], though any mechanistic overlap with PSP is unclear. Analysis of eQTL datasets (in GTEx) suggests that *RPSA* at 3p22.1 and *SKIV2L* near 6p21.1 may also be the causal genes but the tissue-relevant datasets were relatively underpowered.

Aside from identifying additional associated loci and highlighting potential PSP susceptibility genes, we analyzed the polygenic overlap between neurodegenerative diseases, identifying shared heritability with PD and ALS. Curiously, these diseases do not have predominant tau neuropathology, as PSP and other tauopathies do. Typically, PD is associated with aggregation of α-synuclein, and ALS with aggregation of TDP43 and other proteins, while tau pathology is prominent in AD. However, there are known shared genetic risk factors among these diseases. The 17q21.31 haplotype is highly associated with PD, in the same direction as in PSP [44], and SNPs near the *MOBP* gene have been recently associated with ALS [45]. Our results indicate the existence of common neurodegenerative disease pathways even across traditional protein aggregate-based subdivisions, and could potentially lead to effective treatment strategies.

A limitation of the study includes the case-control matching design. While this design allows for matching by array platforms and avoids stratification due to technical artifacts, stratification based on the ancestral differences may be present. The potential for stratification was reduced by strict filtering based on multidimensional scaling to limit the sample to subjects of European ancestry, and linear mixed model methods to further reduce confounding. Combining multiple cohorts as we have done may also reduce the degree of population stratification in the joint sample. Overall, the genomic inflation factor (λ = 1.05) suggested an acceptable level of population stratification. The validity of the results and replication of the original GWAS are further reinforced by the consistency of the identified associations across the multiple platform-matched sub-cohorts.

## Conclusion

Here, we have increased the number of significant genetic risk locus for PSP, an important advance for understanding its pathophysiology. The power of this study to identify novel loci at genome wide significance and a large unexplained heritability suggests that PSP may be highly amenable to genetic association studies in larger sample cohorts using next generation sequencing. Overall, by establishing the genetic correlations of PSP with PD and ALS and identifying novel genome-wide significant and suggestive associations, we shed insight into the mechanisms of neurodegenerative disease.

## Additional files

Additional file 1: Supplementary **Tables S1-S5. **(XLSX 636 kb)

Additional file 2: Supplementary **Figures S1-S5**, Supplementary Methods. (DOCX 8021 kb)

## Abbreviations

AD: Alzheimer’s disease
ALS: Amyotrophic lateral sclerosis
bvFTD: Behavioral variant frontotemporal dementia
eQTL: Expression quantitative trait loci
FTD: Frontotemporal dementia
GWAS: Genome-wide association study
PCA: Principal component analysis
PD: Parkinson’s disease
PSP: Progressive supranuclear palsy
QC: Quality control
SNP: Single nucleotide polymorphism
