# Supplementary material for: Joint genome-wide association study of progressive supranuclear palsy identifies novel susceptibility loci and genetic correlation to neurodegenerative diseases
Source: Mol Neurodegener. 2018 Aug 8;13:41. doi: 10.1186/s13024-018-0270-8 (PMC6083608; doi:10.1186/s13024-018-0270-8)
Supplement: Supplementary file 2 — Supplementary Figures S1-S5, Supplementary Methods. (DOCX 8021 kb) [file 13024_2018_270_MOESM2_ESM.docx]

**Supporting Information**

Chen et al., Joint genome-wide association study of progressive supranuclear palsy identifies novel susceptibility loci and genetic correlation to neurodegenerative diseases

Figure S1

Figure S2

Figure S3

Figure S4

Figure S5

Supplementary Methods

**Figure S1:** Pre-processing steps to create each individual study cohort and the merged joint analysis cohort.


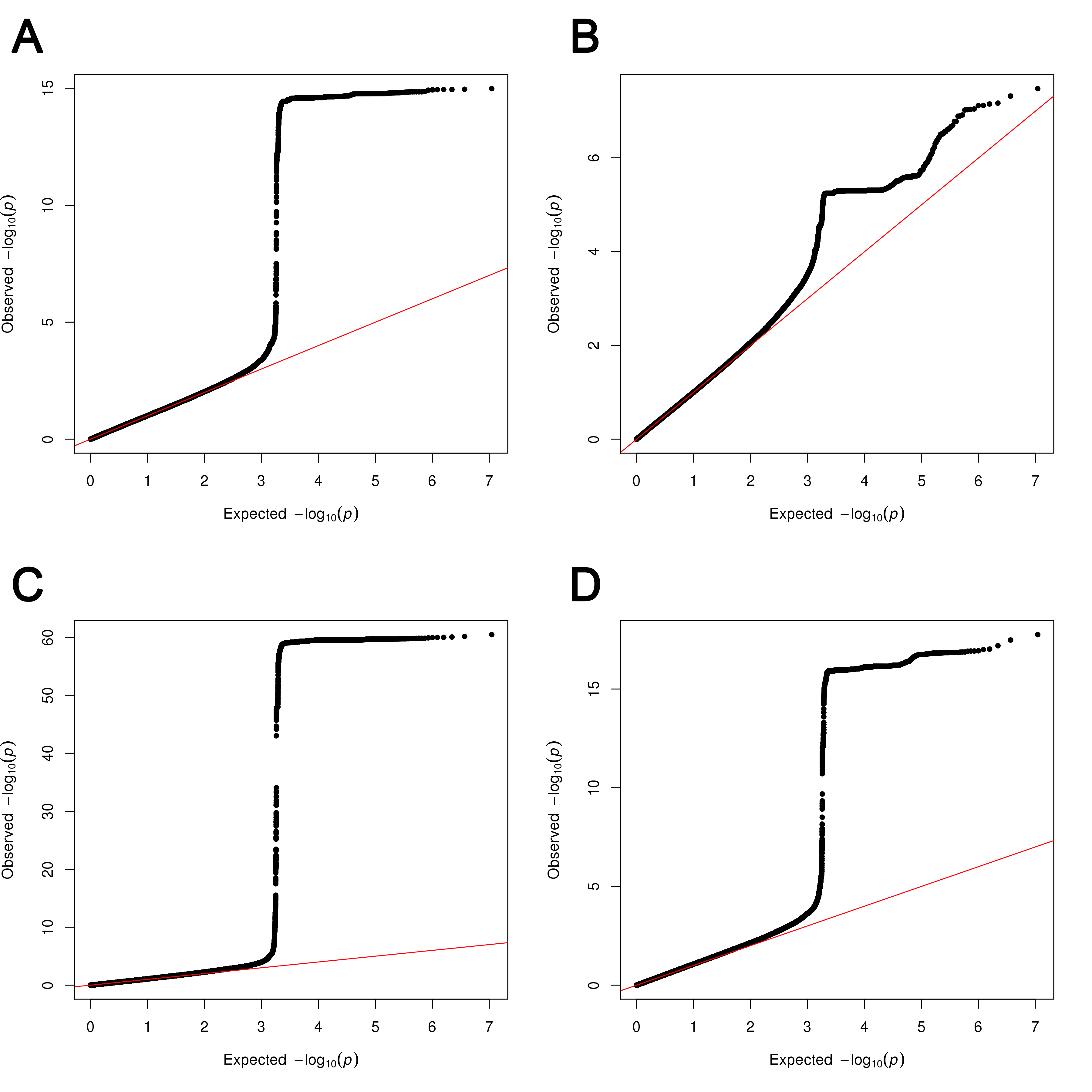


**Figure S2:** Quantile-Quantile (QQ) plots showing the potential for test statistic inflation for all SNPs in A) the UCLA Omni 2.5 cohort; B) the UCLA HumanCore cohort; C) the Hoglinger cohort; and D) the NNIPPS cohort.


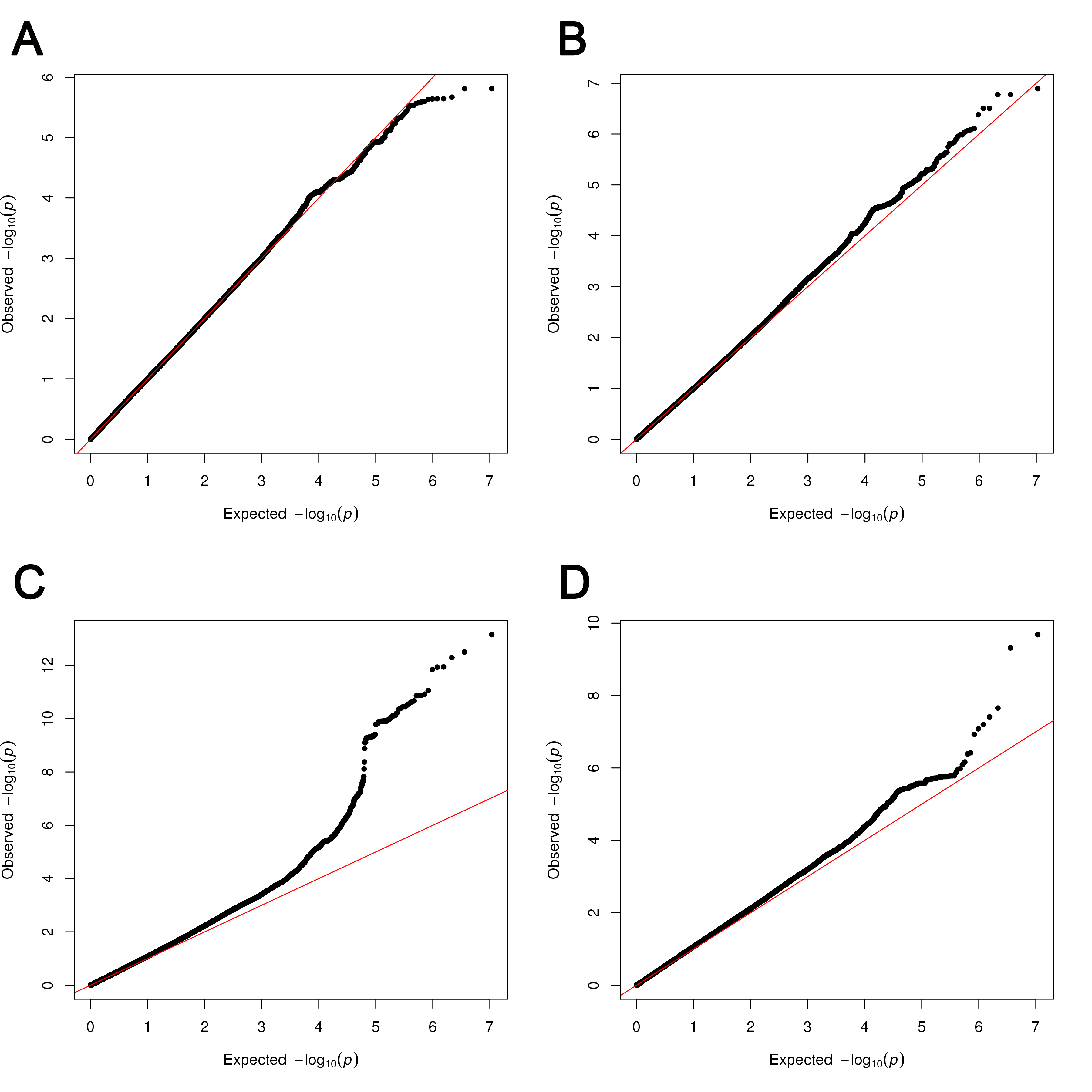


**Figure S3:** Quantile-Quantile (QQ) plots, excluding chromosome 17, in A) the UCLA Omni 2.5 cohort; B) the UCLA HumanCore cohort; C) the Hoglinger cohort; and D) the NNIPPS cohort. Because of the strong association with an extended haplotype region in 17q21, a disproportionate number of associated SNPs reside on chromosome 17.


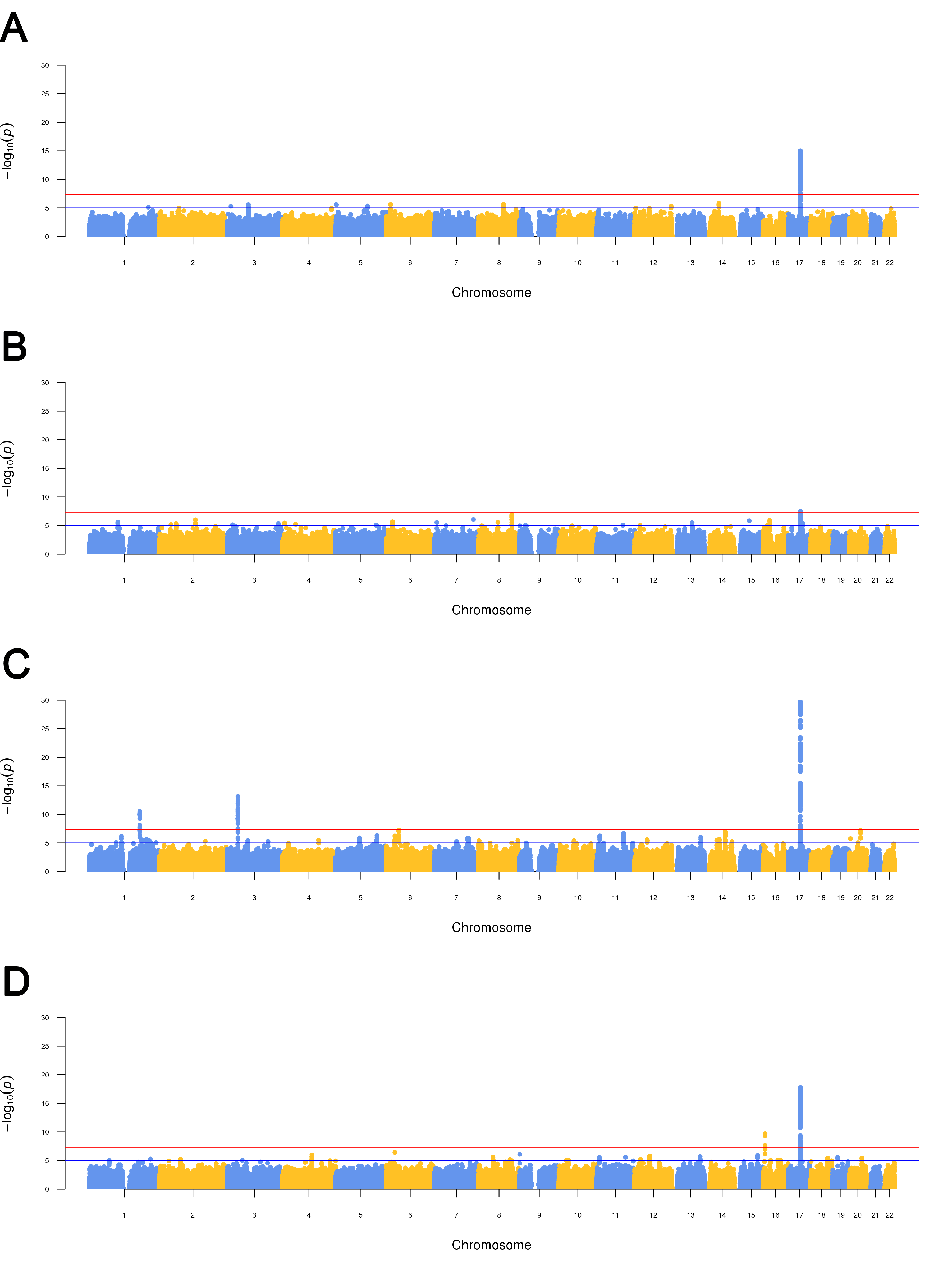


**Figure S4:** Manhattan plot indicating the SNP association *P* values. Genome-wide association results by genomic position in A) the UCLA Omni 2.5 cohort; B) the UCLA HumanCore cohort; C) the Hoglinger cohort; and D) the NNIPPS cohort. The threshold for genome-wide significance (*P* < 5x10^-8^, red horizontal line) is shown. The vertical axis was truncated at *P* = 10^-30^.


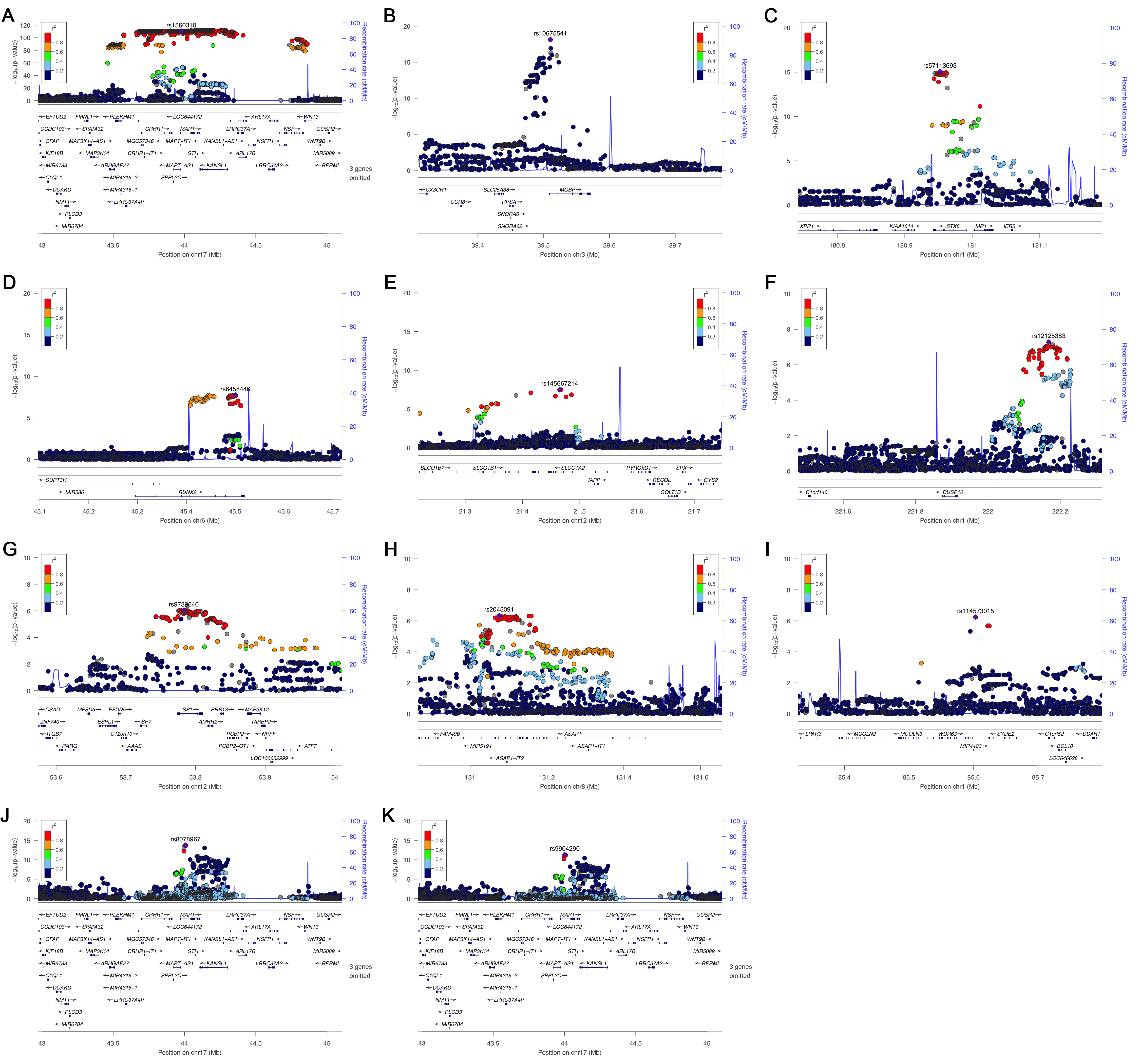


**Figure S5:** Local association results at genome-wide significant and suggestive loci: A) rs71920662 in 17q21.31, near *MAPT*; B) rs10675541 in 3p22.1, near *MOBP*; C) rs57113693 in 1q25.3, near *STX6*; D) rs6458446, tagging rs35740963, in 6p21.1, near *RUNX2*; E) rs145667214, tagging rs7966334, in 12p12.1, near *SLCO1A2*; F) rs12125383 in 1q41, near *DUSP10* in an intergenic region; G) rs9739640, tagging rs147124286, in 12q13.13, near *SP1*; H) rs2045091 in 8q24.21, near *ASAP1*; and I) rs114573015 in 1p22.3, near *WDR63*. The association at 17q21.31 corresponded with an extended haplotype with two non-recombining alleles, H1 and H2. Following regression of the haplotype status, haplotype-independent regional association was found in 17q21.31. K) Similar results are found considering only H1/H1 individuals. Representative SNPs are highlighted. The plots were made with LocusZoom (<http://locuszoom.sph.umich.edu/locuszoom/>).

**Supplemental Methods.**

**Study cohort.**

*UCLA Cohort.* The UCLA cohort was comprised of patients seen at the UCSF Memory and Aging Center and in the Davunetide trial sponsored by Allon Therapeutics (ClinicalTrials.gov trial registration: NCT01110720), with a clinical diagnosis of PSP made using stringent criteria as previously described.^1^ Informed consent was obtained and the study was approved by the IRB at the University of California, San Francisco.

*NNIPPS Cohort.* The NNIPPS (ClinicalTrials.gov trial registration: NCT00211224) and BBBIPPS (French National Health Department Registry Number: DGS N°DGS2006/0524) studies enrolled patients aged between 30 and 80 with an akinetic-rigid syndrome, using validated diagnostic criteria to distinguish PSP and MSA that have been reported in detail previously.^2^ All cases were of European ancestry, recruited from the UK, Germany, and France. DNA was extracted after ethically-approved written informed consent. BBBIPPS used the same NNIPPS diagnostic criteria and was conducted in the same study centres in France.

*Genome-wide association study correlations*

External GWAS were used to identify genetic correlations with PSP. We assessed genetic overlap of PSP with GWAS of other neurodegenerative diseases, including the IGAP AD^3^, behavioral variant FTD (bvFTD)^4^, 23&Me Parkinson's disease (PD)^5^, and Project MinE amyotrophic lateral sclerosis (ALS)^6^, by using GWAS summary statistics. The summary data were converted to a common file type using the "munge_sumstats.py" function in the LDSC software.

The International Genomics of Alzheimer's Project (IGAP) is a large two-stage study based upon genome-wide association studies (GWAS) on individuals of European ancestry.^3^ The summary statistics from stage 1 were used in this study. In stage 1, IGAP used genotyped and imputed data on 7,055,881 single nucleotide polymorphisms (SNPs) to meta-analyze four previously-published GWAS datasets consisting of 17,008 Alzheimer's disease (AD) cases and 37,154 controls (The European Alzheimer's Disease Initiative – EADI the Alzheimer Disease Genetics Consortium – ADGC The Cohorts for Heart and Aging Research in Genomic Epidemiology consortium – CHARGE The Genetic and Environmental Risk in AD consortium – GERAD).

Ferrari et al. performed an international, multicenter two-stage GWAS on patients with frontotemporal dementia (FTD) and healthy controls of European ancestry.^4^ FTD subjects could be further divided into four subtypes: behavioural variant FTD (bvFTD), semantic dementia (SD), progressive non-fluent aphasia (PNFA), and FTD overlapping with motor neuron disease (FTD-MND), as well as a meta-analysis of all subtypes. We used stage 1 association summary statistics in this study. In stage 1, there were 1,634 patients with bvFTD, 361 with SD, 335 with PNFA, 229 with FTD-MND, and 4,308 healthy controls. Samples were genotyped using Illumina Human 370K, 550K, 660K Quad, and Omni Express arrays. Imputation was performed against the haplotypes of the 1000 Genomes Project, August 2010 release using minimac, yielding 6,026,385 SNPs. Analysis of the meta-analysis summary statistics revealed that the trait heritability was not distinguishable from chance, and thus could not be included in further analysis. Of the remaining subtypes, only bvFTD was sufficiently powered.

A GWAS of Parkinson's disease (PD) was performed by 23andMe on patients and controls of European ancestry and included in a meta-analysis of PD GWAS by Nalls, et al.^5^ Genotyping was performed on saliva samples. Samples were genotyped on three genotyping platforms: V1 and V2 platforms were based on the Illumina HumanHap550+ BeadChip, in addition to custom SNPs, totaling ~560,000 SNPs in 3,261 cases and 29,499 controls. These platforms were combined for association analysis because of their high similarity. The V3 platform was based on the Illumina OmniExpress+ BeadChip, with custom content that overlapped the V2 array, totaling ~950,000 SNPs in 866 cases and 32538 controls. Analysis was performed separately from the V2/V3 association. Imputation for each dataset was performed against the haplotypes of the 1000 Genomes Project, August 2010 release using Beagle. These data were downloaded, and meta-analysis of the two datasets was performed using the "meta" package^7^ (https://mathgen.stats.ox.ac.uk/genetics_software/meta/meta.html), --method 1 (inverse-variance method based on a fixed-effects model).

The Project MinE ALS GWAS was performed using 12,577 cases and 23,475 controls, filtered for European ancestry.^6^ Genotyping was performed using the Illumina OmniExpress Array. Summary statistics were downloaded from the Project MinE website at <http://databrowser.projectmine.com/>.

The Psychiatric Genomic Consortium performed GWAS in five major psychiatric disorders: autism spectrum disorder, attention deficit-hyperactivity disorder, bipolar disorder, major depressive disorder, and schizophrenia, in 33,332 cases and 27,888 controls of European ancestry. We used summary statistics from schizophrenia and bipolar disorder cohorts following variant imputation from <https://www.med.unc.edu/pgc/results-and-downloads>.

The GIANT consortium GWAS of height was used as a non-neurological control dataset.^8^ This study included 253,288 subjects genotyped with Affymetrix, Illumina, or Perlegen arrays and imputed against the Phase II CEU HapMap. Summary statistics were obtained from <http://portals.broadinstitute.org/collaboration/giant/index.php/GIANT_consortium_data_files>.

The DIAGRAM consortium stage 1 GWAS of type II diabetes mellitus was used as a non-neurological control dataset. This meta-analysis consisted of 12,171 cases and 56,862 controls, imputed against the Phase II CEU HapMap. Summary statistics were obtained from <http://diagram-consortium.org/downloads.html>.

To estimate the proportion of liability explained by typed genetic variation, disease prevalence values were estimated at 0.0065% for PSP, 5% for AD, 0.015% for bvFTD, 2% for PD, 0.005% for ALS, 1.2% for schizophrenia, 4.4% for biopolar disorder, and 9.3% for type II diabetes, based on reviews at [www.uptodate.com](http://www.uptodate.com).

1. Boxer, A.L., Lang, A.E., Grossman, M., Knopman, D.S., Miller, B.L., Schneider, L.S., Doody, R.S., Lees, A., Golbe, L.I., Williams, D.R., et al. (2014). Davunetide in patients with progressive supranuclear palsy: a randomised, double-blind, placebo-controlled phase 2/3 trial. The Lancet Neurology 13, 676-685.

2. Bensimon, G., Ludolph, A., Agid, Y., Vidailhet, M., Payan, C., and Leigh, P.N. (2009). Riluzole treatment, survival and diagnostic criteria in Parkinson plus disorders: The NNIPPS Study. Brain 132, 156-171.

3. Lambert, J.-C., Ibrahim-Verbaas, C.A., Harold, D., Naj, A.C., Sims, R., Bellenguez, C., Jun, G., DeStefano, A.L., Bis, J.C., Beecham, G.W., et al. (2013). Meta-analysis of 74,046 individuals identifies 11 new susceptibility loci for Alzheimer's disease. Nat Genet 45, 1452-1458.

4. Ferrari, R., Hernandez, D.G., Nalls, M.A., Rohrer, J.D., Ramasamy, A., Kwok, J.B.J., Dobson-Stone, C., Brooks, W.S., Schofield, P.R., Halliday, G.M., et al. (2014). Frontotemporal dementia and its subtypes: a genome-wide association study. The Lancet Neurology 13, 686-699.

5. Nalls, M.A., Pankratz, N., Lill, C.M., Do, C.B., Hernandez, D.G., Saad, M., DeStefano, A.L., Kara, E., Bras, J., Sharma, M., et al. (2014). Large-scale meta-analysis of genome-wide association data identifies six new risk loci for Parkinson's disease. Nat Genet 46, 989-993.

6. van Rheenen, W., Shatunov, A., Dekker, A.M., McLaughlin, R.L., Diekstra, F.P., Pulit, S.L., van der Spek, R.A.A., Vosa, U., de Jong, S., Robinson, M.R., et al. (2016). Genome-wide association analyses identify new risk variants and the genetic architecture of amyotrophic lateral sclerosis. Nat Genet 48, 1043-1048.

7. Liu, J.Z., Tozzi, F., Waterworth, D.M., Pillai, S.G., Muglia, P., Middleton, L., Berrettini, W., Knouff, C.W., Yuan, X., Waeber, G., et al. (2010). Meta-analysis and imputation refines the association of 15q25 with smoking quantity. Nat Genet 42, 436-440.

8. Wood, A.R., Esko, T., Yang, J., Vedantam, S., Pers, T.H., Gustafsson, S., Chu, A.Y., Estrada, K., Luan, J.a., Kutalik, Z., et al. (2014). Defining the role of common variation in the genomic and biological architecture of adult human height. Nat Genet 46, 1173-1186.
